# Actor-center framing on measuring land use conflict visibility

**DOI:** 10.1016/j.mex.2021.101450

**Published:** 2021-07-10

**Authors:** Muhammad Alif K. Sahide, Nurhady Sirimorok, Karno Batiran, Micah Fisher, Bart Verheijen, Mitalia Nonza Sulu, Fatwa Faturachmat, Supratman Supratman, Ahmad Maryudi

**Affiliations:** a Forest and Society Research Group, Faculty of Forestry, Universitas Hasanuddin, Makassar, Indonesia; b Peasant School Network of PAYO-PAYO Indonesia; c Sebijak Institute, Forestry Faculty, Universitas Gadjah Mada, Indonesia; d Department of Urban and Regional Planning, University of Hawaii at Manoa, United States; e Colonial and Global History. Leiden University, the Netherlands

**Keywords:** Conflict visibility, Latent and manifest, Conflict asymmetry, Land use conflict

## Abstract

Land use conflict's visibility assessments are often reserved to descriptive reports in their measurement of a conflict depth. The existing literature has limited the ability to measure the degree of the conflict visibility. In light of this deficiency, we establish a technique to measure the degree of both latent and manifest conflicts using the actor-centered framing. This heuristics approach is focused on the gradation of interaction between conflicted actors by juxtaposing state and local community actors in defending their interest. We measure how state actors deploy policy instruments, mobilizing resources and interventions, and vice versa observing local actors that seek to internalize their issues and interests and getting public attention by building alliances and advocacy. This paper proposes a novel framework for exploring what is implied by latent and manifest tensions between local community and land use government institutions in greater depth. Practitioners will be able to get a better understanding of the conflict visibility, and to develop suitable intervention in conflict in order to reach a manageable situation. This paper also generates possible hypotheses for future research by examining how actors develop and utilize policy instruments for their interest in managing land use conflict.

• Juxtaposing powerless and dominant actors.

• The gradation points of latent and manifest in conflict visibility continuum.

• Actors’ tendencies toward the conflict

## Graphical abstract

**Figure:**
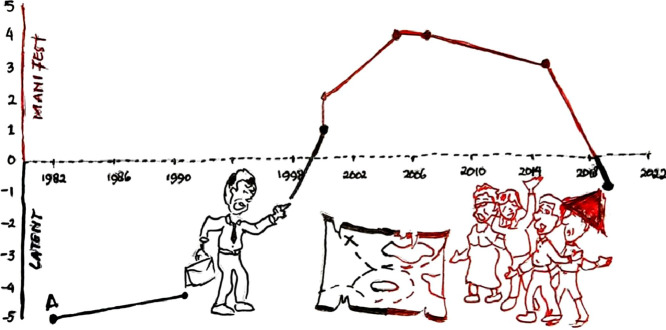

Specifications table

**Table:** 

Subject area:	Social sciences
More specific subject area:	Forest policy and governance
Method name:	Actor-centered Conflict Visibility (AcCV)
Name and reference of original method:	Yusran, Y., Sahide, M. A. K., Supratman, S., Sabar, A., Krott, M., & Giessen, L. (2017). The empirical visibility of land use conflicts: From latent to manifest conflict through law enforcement in a national park in Indonesia. *Land Use Policy, 62*, 302-315.
Resource availability:	Not applicable

## Background

The exploration of land use conflicts, somehow, overlooking the relation between macro political context with the local context. It is especially to global south countries where both scales have significant dynamics which is often influenced by the globally political economic interests [1]. For instance, the national government can be in a transition era to democracy but remains in keeping the corporate interests intact. Thus, researchers need to pay more attention to the multiplicity of the conflict scales at play to minimize the gap between the context scales, i.e., be able to zoom in and out of the scale. Another important issue in studies on land use conflicts is that they tend to provide only two forms of conflict, latent and manifest, with no specific gradation of the latent and manifest [1,2]. Finally, the influence of deliberative approach from the global instruments, which in implementation tend to become apolitical and neglect the power asymmetry [3,4], needs some improvements. Therefore, a new approach is needed to describe multiple levels and depth of conflict, the plurality of interests and power, and at the same time it should be applicable enough to be inducted to the management and intervention of conflict.

## Juxtaposing powerful and weak actors in land use conflict

Land use conflict is discussed in the literature in a variety of ways, from conflict awareness and regulation to conflict mediation and resolution [5,^6^,[1], [2]. To grasp a land-use conflict, one must first explain how it changes in a continuum from a latent to manifest state, and the depth of both states. More specifically, it should show the degree of conflict visibility in a conceptually and empirically measurable way. This conflict visibility measurement approach acknowledges that the conflicts are asymmetric, involving parties with unequal power and status, and protracted, “crossing repeatedly into and out of violence” [7]. The approach aims to provide a theoretical but realistically quick assessment of how conflicts are shifting along the continuum of latent and manifest states, how this exposure defines the role of deployed policy in the conflict, and identify the consequences of interventions.

Under the three-dimension framing (such as substantial, regulatory, and empirical), Yusran [2] provides only two forms of conflict, latent and manifest, with no specific gradation of the latent and manifest. This dual visibility relation is insufficiently evaluated. Political ecologists often describe the land use conflict dynamic without the graphic overview of the visibility ( e.g. Brogden and Greenberg [8] that looking reterritorialization at land use conflict in Arizona or Benjaminsen et al. [9] that analysed the Tanzanian pastoral policy at land use conflict in Kilosa District). We suggest an original approach to enhance the visibility of the conflict depth, by paying closer attention to the details of how players respond to the conflict and the degree of which they gain or lose their position and interest.

This approach aims to develop a theoretical yet more realistic rapid assessment of how conflict shaped itself in latent and manifest gradations, as well as reflect the conflict's interest and circumstance. This approach may provide appropriate intervention over the complex dispute exposure of latent and manifest conflict, which can be repeated on land use conflict by the use of actors-centered framing. This will necessitate a longer time frame; however, the visibility framing would assist the involved actors by allowing them to see a wider time-frame of the dispute [10] and therefore would assist the actors to be aware of the state of conflict.

Aspects to be considered in this approach include actors and their institutions, their priorities or agenda, as well as the degree of information and authority they possess. Actors and their priorities are at the forefront of the dispute agenda. Information and authority are used to exercise influence [11]Conflicts can be dynamic, influenced by a variety of variables such as national policy, law enforcement, deadlock policy, the degree of local community participation, the contested-fragmented and complex interests among actors [12], but by utilizing this actors- interest framing, and considering the asymmetric nature of conflict, we render a heuristic simplification by juxtaposing only two types of actors, such as between government actors and local community actors.

Type A: Actors that are dominant, ordinated, or authoritative. Those players with authority (e.g., government departments, private companies ruling the land) are usually in charge of providing policy instruments (e.g., issuing new policies or enforcing existing ones)

Type B: helpless actor, often a small society that has lost its informational, institutional and commonality control over the land and has become a victim of policy interference, sacrificing its essential needs.

In our definition, we simplify the latent conflict as implies that there is a t competition (fighting over the same resources) but deficient awareness of relevant actors, or the broader public is unaware of the dispute. In this unseen mode, actors A and B might be able to see the disparities in their positions and interest in a land use arrangement, but the discourse is also not receiving public scrutiny. Vice versa, manifest conflict indicates that the conflict has effectively drawn the public attention and could even allow outside parties to directly engage.

## Space power context and Intervention in land use conflicts

In an authoritarian government, the government will effectively deploy policies that oppose the interests of the local population. It is still possible to make the conflict visible, but a militaristic approach and no independent media exposure would scuttle this conflict from the public, and in a real situation weeding out the people from conservation areas, or making a new land tenure arrangement that is totally different from traditional land tenure are easily deployed in this situation without getting conflict manifested in the field. Since there is no mediation and the local population has no channel or force to challenge the government, there is no confrontation in this case. Gaventa [13] constructs this context as the ‘closed space’, where authoritarian agencies close the door for participation.

In the more democratic government, the conflict dynamic is more complex. When the government switches into a more democratic governance, all previous issues will resurface, and the government will be in a vulnerable position. However, the government can still exert power over the situation by delaying policy resolution, hollowing out policy, or even making policy appear to be enforced without proper implementation. The deadlock in legislation, according to Sahide et al. [14] may be an intentional attempt to block local community priorities. This can be seen from the way the government keeps their fundamental interests’ agenda by allowing the policy to be officially announced and enforced without law enforcement or by abusing the flexibility of formal procedure of discretion to disregard local community interests. The government can even switch back into their authoritarian style in managing land use conflict in a democratic system [15,16]. Gaventa [13] names this arrangement as the ‘invited space,' e.g the government provides a selective room for participation and labels these measures as typical ‘initiative’ or even ‘agrarian reform’. It is a space for selective involvement to demonstrate the agencies’ desire to present their ‘good face” for the local community as a seemingly accommodating government. Agrarian reform policy [17] partnership scheme [18] and or co-management [19] would be easily disseminated as a formalistic jargon that only serves to conceal the real macro political interest (for example, influential entrepreneurs, elite actors' investments [20] and infrastructure development [21]).

## Action - reaction of actors: policy instrument politics vs grassroot movement

**Fig. 1:**
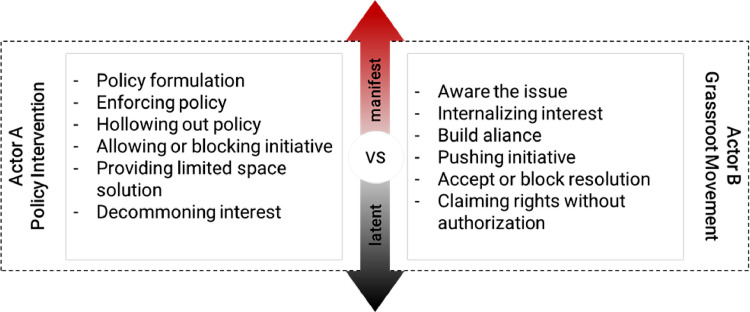
Possible resources of involving actors

As seen in Fig. 1. We provide space for both actors' possibility to take action and observe how opponent actors react and lead to the qualitative gradation of manifest and latent conflicts. Actor A plays a crucial role in land use conflict visibility. Agencies responsible for the land use may control the dispute and include a variety of settlement solutions by policymaking (e.g., policy formulation, policy enforcement, offering co-management). In a particular way, the policy itself can be a source of contention and dispute. Actor A could provide a hollow regulation to keep the conflict latent, or create division among actor B (decommoning interest). According to Sahide et al. [9] a hollow policy creates room for a policy agenda but absolves the agency of the burden of actively carrying out those promises. Actor B has several options following their ability to grasp the real issue that creates conflict. They may start to disseminate the problem to internalize common interest and further build alliances with relevant actors outside of the actors directly affected by the conflict, after which they might be able to push for and/or reclaim local land use interest. This may or may not include legal efforts advocacy.

We used quantitative methods to assess the consistency of actors' behavior in voicing their roles and fighting for their interests. The ranking criteria for this quantification are as follows:

a. A score of 0 means that the actors have begun to attempt to resolve the dispute and recognize their adversaries. Since the actors are aware of the tension, it is always essential for it to become latent or visible. This conflict is contingent on how the actors respond to it.
b. A maximum point is indicated as the strongest core actors’ reaction towards specific policy formulation or enforcement, showing their position and interests openly or empirical accessible
c. A minimum point is indicated as the very closed expression towards specific policy formulation or enforcement, showing their position and interests or interests in limited expressed and even inaccessible
d. Additional 1 point on each of interest action movement (as the representative of their interests’ struggle), is indicated as 1 point manifest movement for the increasing empirical visibility
e Reducing 1 point for each actor lowering their interest standard or hiding their basic interests

Based on these principles, we used a range of -5 (as minimum points) to 5 points (as maximum points), which depended on whether tensions intensified or decreased, and the actors’ interest in performing actions that could attract public concern and involvement. This heuristic measurement of conflict visibility can be seen in Table 1. Table 2.

**Table 1:** The gradation points of latent and manifest conflict: Actor-centered Conflict Visibility (AcCV).

Actors and their interests		Actor's behavior responding each other and the policy instrument	Scoring
Latent	Actor A and B could not consider themselves to be part of the conflict	Actors A starts to develop land use policies that are incompatible with local institutions (Actor B) in terms of land use structure. Actor A is therefore oblivious to the degree to which policy will contribute to land use dispute.	-5
	Actor A hide their interests	Actor A starts to create formal land use institutions silently, they are aware of the new formal institution, but do not understand the purpose of it and do not understand the fully consequences and even detail the legal policy instrument on backing up the new institution	-4
	Actor B start to complain	Land use arrangements of Actor's A continue their existence and raise the attraction of Actor B. Actors B find limited options to express their concerns owing to the policy's common sense that they must endorse.	-3
	Internalizing Actor B interest, while Actor A starts to find a coalition	Actor B starts to discuss internally their position and complaint, but does not have the channel to develop a coalition externally, or energy to push their interest claims. Meanwhile, Actor begins to find alliances.	-2
	Actor A confident with the status quo	Actors A are aware of Actor B's movement, but Actor A also has the momentum to hold the strategy in place by strengthening A's coalition and brodering supporting networks.	-1
Baseline visibility		Each Actor keeps the conflict neither increases nor decreases. Actors B recognizes the impact of Actor A's policy action and defines their opponents and potential coalition. Actors B speculates on Actors A's released policies and begins to engage collaborators to internalize their interests.	0
Manifest	Public begin to identify the conflict	Actors B publicly argues their views and interests attracting broader public attention, but they also lack a solid backup alliance.	1
	Actor B find coalition	Actors B find coalition to advocate B's interest and bring it into mediation channels (both judicial and non judicial form)	2
	Actor A invite for conflict resolution	Actors A openly argue for the purpose of land use policy instruments and open the power space of co-management by offering some limited space for local institution arrangement. (If Actors B lower their standards for basic interests and agree with the consensus and new policy resolution initiative, the visibility will go to the baseline visibility)	3
	Actors continue to fight their interests	Actors A use law enforcement to keep the policies in place because Actors B block the initiative or Actor B ignore the legal standing of Actor A and continue fighting their interests.	4
	Actors take maximum action to keep their own interests and fight for them	Actor A maintains their interests and mobilizes all resources and broadens their institutional back up. While Actor B on the other side maximizes and broadens the grassroot network.	5

**Table 2:** The dynamics of land use conflict visibility in Komara Hunting Park.

Code	Description		Time	Score
	Actor's behavior	Arguments		
A	Central government appointed Komara area as hunting park through Ministry of Forestry regulation No. 147/KPTS-II/1987	Actor A developing hunting area policies without proper consultation (this is during the era of authoritarian regime)	1987	-5
B	BBKSDA had started to claim the Komara Hunting Park as a conservation area, effectively putting the Park under their control, based on the Ministry of Forestry Regulation No. 37/KPTS-II/1997.	Actor A started to use the new and strongest regulation to control the hunting park area contrary with Actor B institution	1997	-4
C	Cakura village's residents held a demonstration in front of the Takalar Regent office while bringing the tax notification letter (SPPT, *Surat Pemberitahuan Pajak Terutang*) as evidence of their property	Actor B publicly convey their own interest to claim their property land	June 2002	1
D	The residents sued the legal standing of the Komara Hunting Park to the High Court of Takalar Regency and they won. Meanwhile BBKSDA appealing this decision to the Supreme Court	Actor B had a coalition with the Regency government to advocate the Actor B's interest	July 2002	2
E	Supreme Court decided that BBKSDA won the land use dispute that the Komara Hunting Park formally be a conservation area under BBKSDA control	Actor A use the Supreme Court decision to strengthen their position and claim, and continuing their policy implementation in the Komara Hunting Park area	2007	4
F	The residents continue with their livelihood activities	Actor B shows their disappointment with the court decision and by still occupying the land for livelihood. They claim their rights without authorization	2009	4
G	BBKSDA collaborated with the village government carrying out several info disseminations on the park, workshops, and empowering the community to take benefit sharing of the hunting park area.	Actor A opened a chance for a co-management with Actor B.	2017	3
H	BBKSDA establish three community groups of deer breeding and give the chance to the residents to take advantages from the hunting park area through utilization of non-timber products	Actor A and Actor B remain	2020	0
I	Regency government support the BBKSDA to manage a hunting park to support its interest on local tourism business as long as BBKSDA supporting local people empowerment	Actor A successfully steals the B's main coalition. Actor A had the momentum to lobby the new Regent of Takalar to support their both interests	2021	-1

**Fig. 2:**
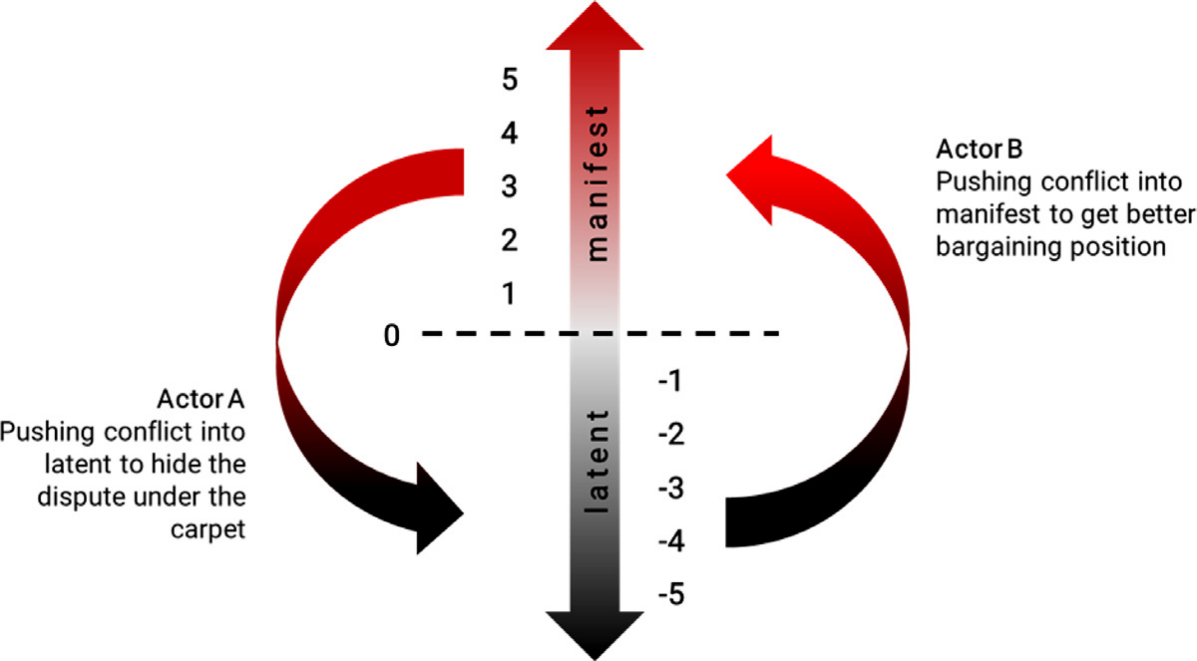
Conflict visibility continuum and actors tendencies toward the conflict

Conflicts between parties are typically plural and sustained, they coexist and/or shifting from one to another, they cannot be eliminated altogether. Therefore, the more powerful actors (Actor A) tend to push their conflict to the latent state to minimize the risk of being exposed to the level they are able to manage, while on the other hand the weaker actors (Actor B) tend to expose their conflict to gain alliance and/or attract public scrutiny which can increase their bargaining position (Fig. 2). In other words, while Actor A tends to limit participation, Actor B tends to attract more participation. Each of the points of conflict gradation can be stalled in a status quo, they can also jump relatively fast to any point of gradation along the continuum, depending on the actions taken by parties involved.

## What empirical material to search for?

For the land use conflict scholars, we provide several hints of relevant data that researchers could collect. The interview can find the ‘trim tab” [22] or key resource persons to navigate the understanding of the conflict visibility. If researchers use observation, they can start to understand actual issues as seen from both sides, what information is held and distributed, and observe further action and reaction of policy as well as the grassroot movement. Below are the common hints.

### Zoom out: General context

• Identify the dynamic of the global political economy that directly (and indirectly) influence land use management, intervention, and current global instrument induced to conflict mediation
• Identify the outlook of the current government system, whether it tended to be authoritarian or democratic
• Identify the forms of social institutions and local traditions in the conflict area.
• Explore the history of community land use and management, along with its dynamics and change.
• Identify the reasons for the emergence of the conflict and how each conflict was resolved through the bureaucratic mechanisms that were working at that time

### Zoom in: local context

#### Grasp visibility from actor A position

• Seek to understand any policy related to the studied conflict.
• Explore regulation documents related to the conflict.
• Identify government agencies involved in the conflicts as well as understanding main tasks and authority of the agencies.
• Examine the methods and strategies used by the government actors to handle and/or contain the conflict so it does not spread and catch critical attention of the wider public.
• Identify main interests of the government related to the land under conflict.
• Identify parties supporting the government in handling the conflict and their cooperation mechanism.
• Explore the extent of the third-party involvement in the conflict.
• Explore the contribution of the third party in the conflict dynamics, whether they help to make the conflict become more latent or manifest.

#### Grasp visibility from actor B position

• Observe whether the people realize that they are in a conflict situation.
• Explore whether the people understand the dynamic of the conflict that involves them.
• Identify the basis of the people's claim on the land that they consider their right.
• Identify the responses of the people towards the government attitude and/or action related to the conflict.
• Explore the causes of people's failure to claim their rights to their land.
• To find out whether the people involve other parties to support them to win their claim over the land.
• Identify why the people seek for help or involve other parties in their effort to win the claim over the land.
• Identify who and under what capacities the supporting parties are helping the people in the conflict resolution process.

## A bundle of hypothesis on measuring land use conflict visibility

We provide several potential hypotheses by using this approach. The hypotheses can help to identify which actors win and lose at a certain point along the conflict continuum

a) This approach to determine the points of conflict visibility is not devised to delve into the dynamic nor the type of emerging resolutions of the conflict, but typically there are three types of resolutions of conflicts, which means that the conflict situation is subside into a manageable one, albeit it not means that the situation can reheated).
  1. Actor A wins: Actor A gets what they desire, suppress the Actor B resistance, or shift the issue that generated the conflict to another one, or move the conflict to be coped with by other parties.
  2. Actor B wins: Actor B gets what they desire, although sometimes temporary.
  3. Draw: Each party involved gets what they desire, at least to the degree that they can tolerate (compromise), and this state of affairs continues for a relatively long period of time (we could call it a status quo).
b) Draconian instruments such as law enforcement will reveal the conflict on the surface and lead the actors involved to reconsider their interests
c) There are conditions where everyone is aware of the conflict but suppresses it at the latent state for a relatively long period of time. In other words, the conflict is an open secret, visible but fails to manifest. This state may stem from cultural attitude toward conflict or history of suppression of an authoritarian regime, or mix between them. The approach may fail to grasp this type of state. One way to get around this problem is to take a longer time-frame in examining the conflict, as the latent state may represent a certain time-frame that only matches the research scope.
d) Regarding the fragmented and plural interests of internal local community, Actors B (i.e., villagers who live directly within the village's territory, versus those who live outside it), finding a common concern can be easy or difficult, depending on the quality of relationship among actors within a group or the degree of (mutual) opponent pressure.
e) The nature of land use conservation-oriented bureaucracy that tends to hide their conservation interests by expanding their interpretation on formal mandates that allow them to include production and social mandates.
f) Local agencies and third parties (such as NGOs) can make use of a dispute to achieve their own interests (i.e., political electability for district elections for local agencies, or achieving claim of successful program for third parties)

## A case illustration: A simple example of practical application of the method

**Fig. 3:**
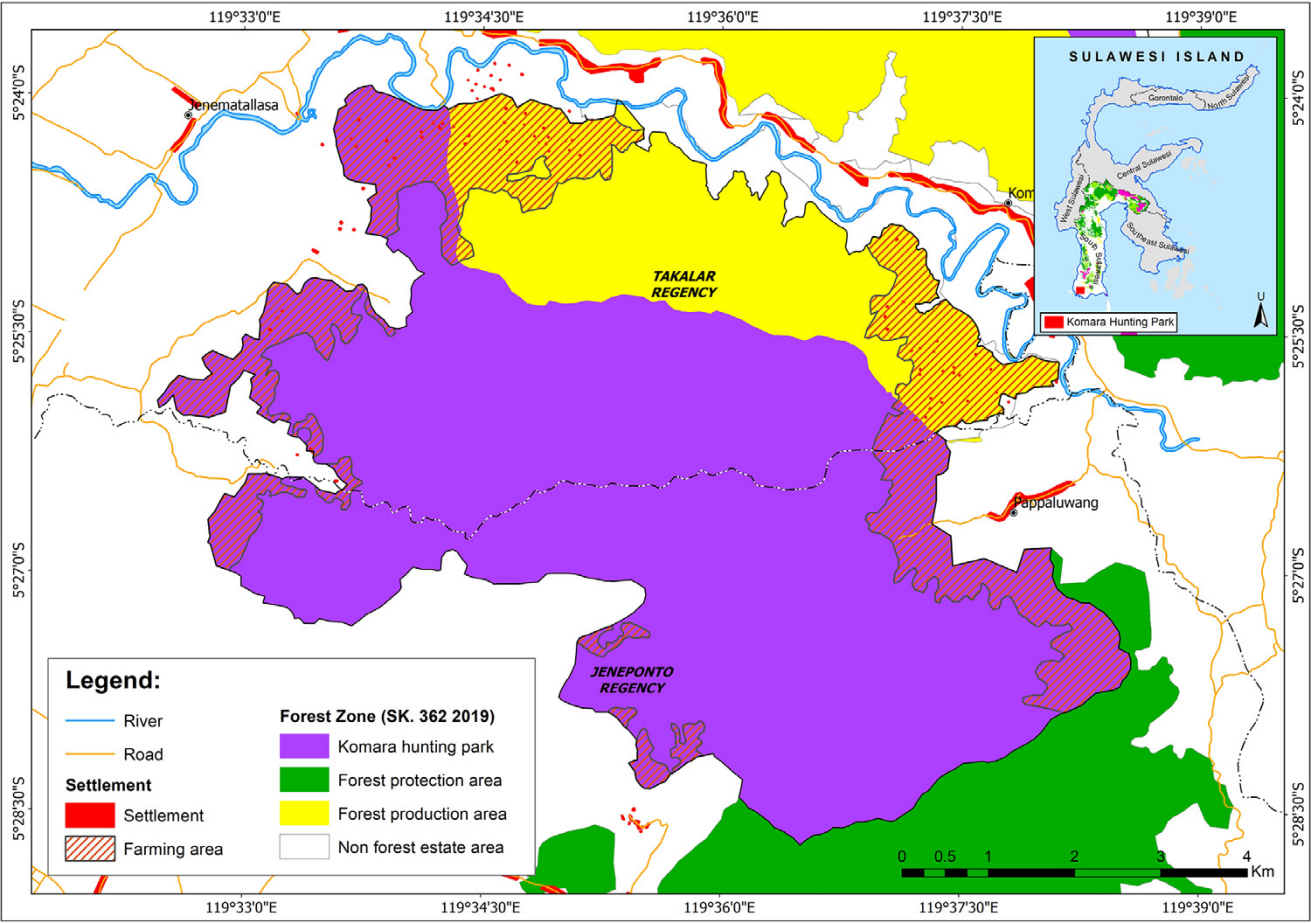

In order to illustrate this AcCV method, we provide a brief pilot example that summarizes a conflict situation in - a hunting park in Indonesia , with the hope that it would encourage others to apply the approach. It is a land use conflict involving the Komara Hunting Park *(Taman Buru Komara*) (see Fig. 3) in the Indonesian province of South Sulawesi. Actor A is represented by the South Sulawesi Natural Resources Conservation Agency Center (*Balai Besar Konservasi Sumberdaya Alam Alam Sulawesi Selatan*, BBKSDA), which manages the park. Actor B is represented by the Cakura village community, who oppose the park's establishment.

The Komara Hunting Park has a long and complicated history, which still influences the identity of the conservation area. In the sixteenth and seventeenth century the area was used as an elite deer hunting area by the aristocratic kings of Gowa, Tallo and Takalar. The local community's interest in the hunting activities has always been restricted by the elite. After the Indonesian Republic was formed in 1945, the community abandoned hunting altogether and instead emphasized farming as a means of subsistence. However, the historical dimension of aristocrats hunting in this area was the primary motivation behind singling out this area as a conservation area with the purpose of establishing a Hunting Park in 1987.

During the authoritarian New Order regime (1965-1998), the vast majority of Indonesia's landmass was designated as forest areas. These measures ignored community social interests for the most part and relied heavily on physical criteria (such as precipitation, 'slope', a unique landscape, protection of endemic species, and soil types) in determining where areas such as forest and conservation areas should be located. Despite the fact that the local community formulated its own standards and criteria for managing land distribution, the community appears to incline to more repressive practices in relation to redistribution and development of land. There have been several structural changes since the fall of the authoritarian regime and the beginning of the 'Reformation' in 1999 and these have continued until today. However, the presence of the historically grown elite culture of corporatism is a threat and hinders addressing the fundamental issues.

Although decentralization of the forestry sector has been effectively accomplished on a district and provincial level[23], conservation areas continue to be under the jurisdiction of the central government, including the Komara Hunting Park area. As a result of international pressure on the recognition of indigenous and local rights and the process of participation of local communities, and, on the other hand, the high frequency of agrarian land conflicts, the government decided to open the gate for recognition of access and agrarian reform. This however brought old and new land use conflicts to the surface. To present this complicated situation of land use, in this case illustration, we stipulate that Actor A wins due to backing of centralized (governmental) power to carry out the conservation mandates. Thereby using tools as: 1) coercion power (law enforcement, to push the conflict to serve their interests) and 2) conservation partnership incentives. These two powerful instruments can push the conflict into newer (and latent) forms as explained in detail below.

**Fig. 4:**
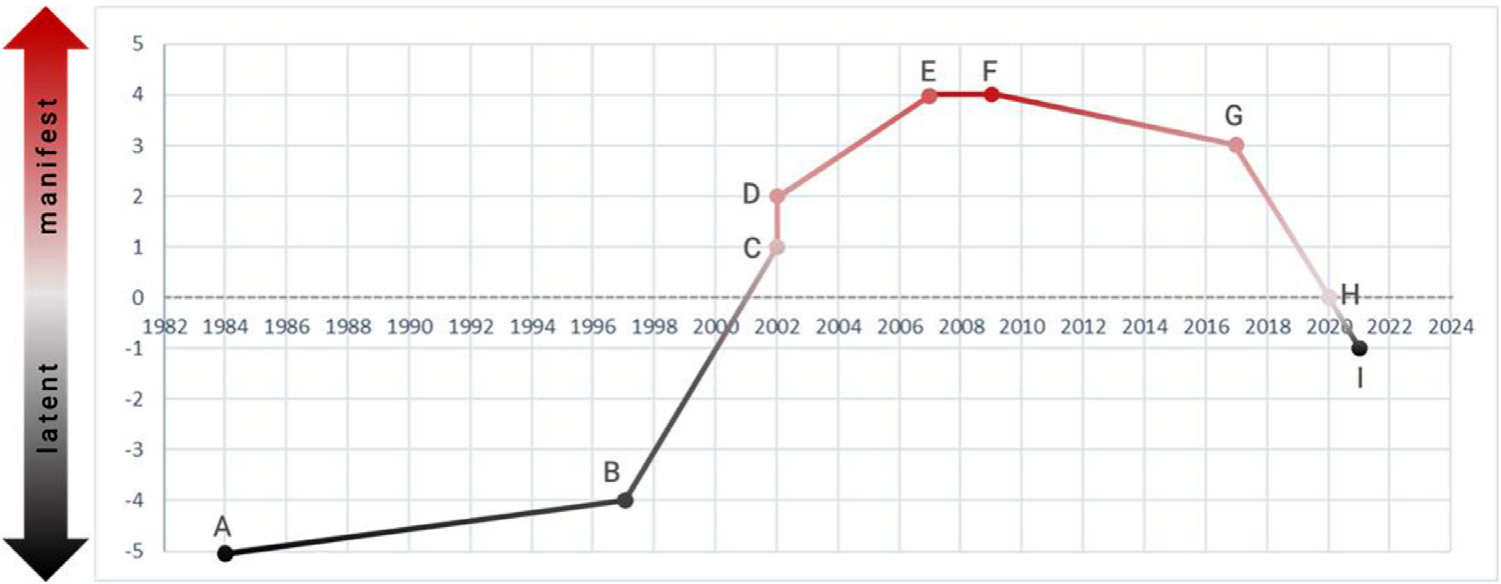
The histogram of the conflict visibility in the Komara hunting park

The land use conflict visibility in the Komara hunting park which is described above can be seen in the histogram (see Fig. 4).

The trendline in the histogram above shows how over time the conflict gradually increased from latent to manifest, and back to latent. At the beginning, in 1984, the appointment of forest area had been carried out haphazardly by the central government without considering the local context. At this point, the conflict was in scale -5 where the residents did not have any knowledge nor power to wage any resistance. This resulted in the establishment of Komara Hunting Park in 1997 without involving the community in the area. Since that time, the community has attempted to claim their land rights and push the conflict to manifest form. Furthermore, in 2002, it was shifted to take more attention in the local context and show how the residents, through demonstration and lawsuit, have struggled to maintain their claim and push the conflict into manifest Here, the conflict reaches scale 2 as it shows how the villagers openly face the BBKSDA, while the more powerful state agency also uses law enforcement to legitimize their standing position.

For almost two decades, from 2002 to 2020 the conflict stayed in the manifest dimension where the residents had several momentums to push the conflict further up the manifest dimension: from scale 2 to 4. However, the manifest conditions are not steady enough because in return the BBKSDA carry out efforts to fight back for their interest by forming a coalition with the village government. In addition, the BBKSDA also opened the chance to co-manage the park with the villagers (invited space) in order to retard the conflict. Because of that, BBKSDA was able to control the momentum and push the conflict into latent form. In the end, as we can see, the conflict ended in the latent dimension because the BBKSDA successfully made a coalition with the regency government who previously was the main alliance of the village residents. Thus, the conflict, as of today, returns to the scale -1.

## Advantages and limitations of the AcCV method

After translating our AcCV method into a case, we found that the method is successfully tested with a case that has clear juxtaposing interests and positions from government institutions (as Actor A) and local community (as Actor B). The advantage of using this method could be to get a deeper insight into the conflict dynamics of the local community without overlooking the broader context like global changes and its political economy dynamics. It can 'zoom out' and 'zoom in' the scales of conflict at the same time by assigning every conflict moment into the manifest and latent dimensions. The framework would result in a gradation and historiography of the conflict dynamics. Furthermore, the histogram will illustrate a trendline of conflict so that we can see how the conflict changes in visibility gradation over time and conveniently knowing the depth of the manifest and latent dimensions. The AcCV method has a limitation when the conflict has other coalition(s) rather than only A's and B's coalition. In this case the AcCV should be modified.

## Declaration of Competing Interest

The authors declare that they have no known competing financial interests or personal relationships that could have appeared to influence the work reported in this paper.
